# Pro-cognitive drug effects modulate functional brain network organization

**DOI:** 10.3389/fnbeh.2012.00053

**Published:** 2012-08-28

**Authors:** Carsten Giessing, Christiane M. Thiel

**Affiliations:** Biological Psychology Lab, Institute of Psychology, University of OldenburgOldenburg, Germany

**Keywords:** cholinergic, noradrenergic, topology, graph, complex network, nicotine, fMRI, imaging

## Abstract

Previous studies document that cholinergic and noradrenergic drugs improve attention, memory and cognitive control in healthy subjects and patients with neuropsychiatric disorders. In humans neural mechanisms of cholinergic and noradrenergic modulation have mainly been analyzed by investigating drug-induced changes of task-related neural activity measured with functional magnetic resonance imaging (fMRI). Endogenous neural activity has often been neglected. Further, although drugs affect the coupling between neurons, only a few human studies have explicitly addressed how drugs modulate the functional connectome, i.e., the functional neural interactions within the brain. These studies have mainly focused on synchronization or correlation of brain activations. Recently, there are some drug studies using graph theory and other new mathematical approaches to model the brain as a complex network of interconnected processing nodes. Using such measures it is possible to detect not only focal, but also subtle, widely distributed drug effects on functional network topology. Most important, graph theoretical measures also quantify whether drug-induced changes in topology or network organization facilitate or hinder information processing. Several studies could show that functional brain integration is highly correlated with behavioral performance suggesting that cholinergic and noradrenergic drugs which improve measures of cognitive performance should increase functional *network integration*. The purpose of this paper is to show that graph theory provides a mathematical tool to develop theory-driven biomarkers of pro-cognitive drug effects, and also to discuss how these approaches can contribute to the understanding of the role of cholinergic and noradrenergic modulation in the human brain. Finally we discuss the “global workspace” theory as a theoretical framework of pro-cognitive drug effects and argue that pro-cognitive effects of cholinergic and noradrenergic drugs might be related to higher network integration.

## Introduction

In the recent years there has been an intensive debate on how to take advantage of brain imaging techniques, in particular functional magnetic resonance imaging (fMRI) to investigate the role of neurotransmitter systems in the human brain. fMRI measurements basically capture blood oxygenation within brain regions via the so-called blood oxygen level dependent (BOLD) contrast which was shown to be related to the processing of neuronal activity within brain regions (Logothetis, 2002). Pharmacological fMRI approaches usually use an acute drug challenge before volunteers undergo a cognitive task inside the scanner and subsequently compare task-related activity between placebo and drug in every single voxel in the brain. Hence, most pharmacological fMRI studies to date have analyzed neurochemical modulation of focal brain activity that is induced by a specific task.

Recently, an increasing number of neuroimaging studies have made use of graph-theoretical measures to interpret the interactions of brain regions and to model the brain's parallel and distributed mechanisms of information processing (Singer, 1986, 2009; Shinkareva et al., 2008). Thereby, the brain is analyzed as a complex network of interconnected processing nodes whose overall behavior is determined by more than the sum of its parts (Bassett and Gazzaniga, 2011). Some pharmacological studies investigated effects of drugs on functional connectivity and the functional interaction between brain regions, but only few of them used graph theory and complex network analysis to analyze drug-induced changes in brain network topology. Within the current article we discuss the advantage of graph-theoretical and complex network approaches for analysis of human pharmacological fMRI data over classical task-activation and functional connectivity analyses with specific emphasis on the noradrenergic and cholinergic neurotransmitter system.

## Cholinergic and noradrenergic drug effects on brain activations

The noradrenergic system has often been studied in relation to attention and recently in relation to cognitive control (Coull et al., 2001, 2004; Chamberlain et al., 2009; Graf et al., 2011) while studies in the cholinergic system focused on modulation of attention- and memory-related brain activity (reviewed in Bentley et al., 2011). For example, Coull et al. (2001, 2004) provide evidence that the α2 agonist clonidine reduced neural activity in parietal and prefrontal cortices, the location of activity reduction was however dependent on the attentional subcomponent investigated and on the underlying level of arousal. Prefrontal and parietal activity reductions with reduced noradrenergic neurotransmission are in contrast to many findings with the cholinergic agonist nicotine. Here, increasing cholinergic neurotransmission led primarily to reduction of neural activity in several brain regions including prefrontal and parietal cortex (Thiel et al., 2005; Giessing et al., 2006; Thiel and Fink, 2008; Hahn et al., 2009). Again, the locations of the modulatory effects of the drug were dependent on the attentional subcomponent investigated, underlining the frequent finding that the location of drug effects in pharmacological fMRI studies are task-specific (Hahn et al., 2009).

Even though existing pharmacological fMRI studies which have used standard data analysis approaches have increased our understanding on how specific neurotransmitter systems modulate specific cognitive functions and focal neural activity in the human brain, a comparison of neurotransmitter systems may benefit from an analytical approach that goes beyond specific local, drug-induced changes and addresses the question how drugs influence global network topology and the organization of brain networks. In contrast to existing approaches to investigate drug effects on brain networks, graph theoretical analyses provide a wide range of analytical tools to precisely describe also subtle, widely distributed drug effects on functional network topology. Thereby, these changes in network topology are directly related to the network's general efficiency and capacity to process information (Bassett and Bullmore, 2006; Achard and Bullmore, 2007). Thus, combining pharmacological imaging with graph analysis offers a new method to understand the biological basis of drug effects on information processing and cognition.

## Drug effects on endogenous brain modes

Changes in BOLD signal due to cognitive or motor tasks are rarely more than 5–10% in comparison to resting state and have only small influences on overall activation levels (Rao et al., 1996; compare also Burton et al., 2004). There is strong evidence that trial-to-trial variability in the magnitude of event-related BOLD signals unrelated to changes of the environment has functional relevance for human perception and performance. Prior studies have documented that the variability in BOLD signal independent of changes in task or stimulus input is correlated with the individual behavior in an Eriksen flanker task (Mennes et al., 2011), individual perception of visual and auditory stimuli (Hesselmann et al., 2008a,b; Sadaghiani et al., 2009), working memory performance (Pessoa et al., 2002), and the preferred problem solving strategy (Kounios et al., 2008). Fox et al. (2007) investigated task-unrelated spontaneous activations within a simple finger tapping task and found that task-unrelated spontaneous activity predicts a significant fraction of BOLD signal variability in brain regions directly related to task processing. They suggested that task-related and spontaneous activations are linearly superimposed and that both in combination contribute to the individual behavioral outcome. Burton et al. (2004) even suggest that the brain largely operates intrinsically and that sensory input modulates intrinsic processes rather than determining brain functions *per se* (compare also Buckner and Vincent, 2007).

It is a common finding that behavioral effects of drugs differ inter- and intraindividually (Bondy, 2005). The study by Coull et al. (2004), which reported that effects of clonidine were dependent on arousal level, supports the view that endogenous brain activity does not only impact on stimulus-evoked neural activity but also on the way drugs modulate this stimulus-evoked activity. Further, there is also a wealth of evidence from behavioral studies that interindividual variation of endogenous brain modes may explain the individual variability observed in many drug studies. For example, only a fraction of patients with Alzheimer's disease benefit from acetylcholineesterase inhibitors (Mehta et al., 2005) and the norepinephrine reuptake inhibitor desipramine showed different neural and behavioral effects in the forced swim test in rats with high vs. low novelty-seeking behavior (Jama et al., 2008). The variability of effects of cholinergic and noradrenergic drugs were shown to depend on a variety of factors such as genetics, gender, or cognitive performance levels (Perkins, 1999; Perkins et al., 1999; Newhouse et al., 2004; Kabbaj et al., 2007; Winterer et al., 2007). Thus, there is a huge range of inter- and intraindividual behavioral variability which suggest a complex interaction between *exogenous*, task-related neural processes and *endogenous*, self-organized personal characteristics or brain states (Reed, 1998; Fox et al., 2006; MacDonald et al., 2006; Adelstein et al., 2011).

To date, the majority of pharmacological neuroimaging studies ignore these endogenous brain states and analyze drug induced changes of task-related neural activity (Furey et al., 2000; Lawrence et al., 2002; Kumari et al., 2003; Bentley et al., 2004; Thiel et al., 2005; Giessing et al., 2006). However, task performance is affected by neural networks directly involved in task processing as well as brain modes which reflect longer-lasting endogenous states (Greicius and Menon, 2004; Buckner and Vincent, 2007) and it is reasonable to assume that drugs will interact with both. First evidence for this interaction was also found on neuronal level. Activity of both, cholinergic and noradrenergic neurons can be categorized into a tonic, task-unrelated, and a phasic, task-related mode which were shown to interact (Devilbiss and Waterhouse, 2004; Parikh and Sarter, 2008). Thus an investigation of drug effects on both, task-related and task-unrelated BOLD signals will add valuable information on how drugs impact on intrinsic and stimulus induced neural activity. A few studies have already started to analyze drug effects on endogenous functional connectivity during resting state conditions.

## Measuring drug induced changes in topology or functional brain organization

### Effects of cholinergic and noradrenergic drugs on functional connectivity

There are now many fMRI studies which investigate functional or effective connectivity between brain regions during task performance or in the resting state. Most of these analyses have tried to investigate the linear or non-linear functional interactions between predefined brain regions and used different measures of statistical dependency like simple linear or partial correlations, mutual information, Granger causality, or coherence (Smith et al., 2011).

Analyses of functional or effective connectivity have been only used in few pharmacological fMRI studies. We here shortly summarize the existing studies in the cholinergic and noradrenergic system. Wink et al. (2006) investigated the muscarinic receptor antagonist scopolamine on functional connectivity in a resting state condition and focused their analysis on connectivity of the hippocampus with six, large brain regions including temporal, parietal and frontal cortex. They found that scopolamine enhanced the connectivity between the frontal cortex and hippocampus, brain regions which were also affected by ageing. Effects of the cholinergic agonist nicotine have also been investigated in the resting state. Tanabe et al. (2011) focused their analysis on brain regions belonging to the so-called default mode network and on extrastriate regions. They provide evidence that nicotine reduced neural activity in the default mode network, and increased activity in the extrastriate resting state network in non smokers. The coupling between the default mode network and an executive resting state network was analyzed in smokers under placebo and nicotine by Cole et al. (2010). Their findings show both, nicotine-induced increases and decreases in coupling of those two networks with different brain regions. Others have focused their connectivity analyses on brain regions such as the cingulate cortex and have shown enhanced functional resting state connectivity of the cingulate cortex with several fronto-parietal brain areas under acute nicotine (Hong et al., 2009). A different approach was taken by Balsters et al. (2011) who combined EEG/fMRI with donepezil administration to investigate cholinergic modulation of oscillatory brain activity which was then related to changes in the BOLD signal. Behaviorally donepezil impaired performance. Neurally drug effects were evident as reductions in alpha and increases in beta and delta power. These maladaptive oscillatory changes were associated with BOLD signal changes within the hippocampus, fronto-parietal brain regions and the so-called default mode brain network.

While connectivity studies in the cholinergic system were exclusively performed in the resting state, most of the studies on the noradrenergic system analyzed changes in connectivity during task performance. Wang et al. (2011) used an effective connectivity analysis to assess the effects of the selective noradrenaline reuptake inhibitor reboxetine in stroke patients within a finger tapping task. They found that reboxetine increased the connectivity between supplementary motor areas and the primary motor cortex. Increases in effective connectivity after reboxetine were also reported in healthy volunteers in a visuomotor control task. Here regions showing increased connectivity were the right visual, intraparietal and superior frontal cortex (Grefkes et al., 2010). McCabe and Mishor (2011) investigated the effects of reboxetine within a resting state condition and focused their analysis on regions of the limbic system. Their data provides evidence that reboxetine reduced the striatal–orbitofrontal cortex connectivity.

Hermans et al. (2011) analyzed the effects of stress and of propranolol, a beta-adrenergic receptor blocker, and found that propranolol reduced functional connectivity in a salience network but not visual network during subjects saw aversive cinematographic material and feared mild electrical shock. Coull et al. (1999) analyzed the effects of clonidine (a2 adrenoceptor agonist) on effective connectivity during a rest condition and during the performance of a visual attention task. During the rest condition they found decreased connectivity between the frontal cortex to thalamus and in pathways to and from visual cortex. In contrast, during the attentional task, clonidine increased the functional connectivity from and to the parietal cortex. These findings resemble the opposing effects of reboxetine found by Wang et al. (2011) and Grefkes et al. (2010) during task performance as opposed to those found by McCabe and Mishor (2011) in the resting state and indicate again the necessity to differentiate between effects of drugs during task performance vs. rest.

Due to the diversity of the administrated drugs, task conditions and applied analysis techniques which often focused on certain brain regions only, it is difficult to integrate the reported results. Furthermore, drug-induced increases or decreases of functional connectivity in specific brain regions can induce quite different changes in functional brain topology and it is unclear whether these changes improved or hindered the brains' capacity for information processing.

### A new approach: brain network topology

Recently, an increasing number of studies extended connectivity analyses by using graph-theoretical measures (see Box 1) to interpret the interactions of brain regions (Achard and Bullmore, 2007; Xia and He, 2011). In contrast to the above approaches complex network- or graph analyses often investigate the whole host of functional connections of the entire brain to describe the topology or organization of functionally connected brain networks. Thereby, the brain is analyzed as a complex network of highly interconnected processing nodes. The nodes often represent anatomically-defined brain regions and edges embody the functional interaction between them. In contrast to normal functional connectivity analyses, graph theory provides a full range of measures to describe drug-induced changes in topology. Thus, with graph theoretical measures it is possible to detect not only focal, but also subtle, widely distributed drug effects on functional network topology. Most important, graph theoretical measures also quantify whether drug-induced changes in topology or network organization facilitate or hinder information processing (Bassett and Gazzaniga, 2011) and thus provide a biological explanation for drug effects on behavioral performance. For example, while Furey et al. (2000) hypothesized in their seminal study that cholinergic neurotransmission increases the efficiency of information processing based on focal activity within extrastriate and prefrontal cortex, a graph analytical approach would enable to quantify processing efficiency with mathematical measures. Currently few studies have used graph and complex network analysis to investigate drug effects on functional brain networks.

Box 1 **Essential principles of graph theory.**

The basic principles of graph theory have been described elsewhere (He and Evans, 2010). In short, the approach can be used for structural MRI, diffusion MRI, functional MRI, and EEG/MEG. For resting state fMRI data graph network analysis includes the following steps (Poldrack et al., 2011, p. 156):

1. *Brain parcellation and data extraction*: The brain is parcellated in different brain regions and the fMRI time course is extracted from each brain region (Zalesky et al., 2010). Each brain region is represented by one node.
2. *Compute the functional connectivity and network adjacency matrix*: A measure of statistical dependency is computed for each pair of node to estimate the strength of relation between nodes. In simplest case an *n* (number of nodes) x *n* correlation matrix is computed to estimate the adjacency between nodes. In many studies, the adjacency matrix is thresholded to exclude edges which reflect noise and to receive binary entries representing the presence or absence of an edge between two nodes.
3. *Characterize the network topology*: Based on these binary adjacency matrices the network graphs are visualized and graph-theoretical measures which describe the topology of the brain network are computed and compared between different groups or conditions.

***Glossary***

*Complex system*: A system of interconnected parts whose overall behavior is more than the sum of its parts and cannot be predicted by its individual elements (compare also Bassett and Gazzaniga, 2011).

*Custering coefficient*: Networks with high clustering are characterized by many connections between the nearest neighbors of any given node.

**Figure 1:**
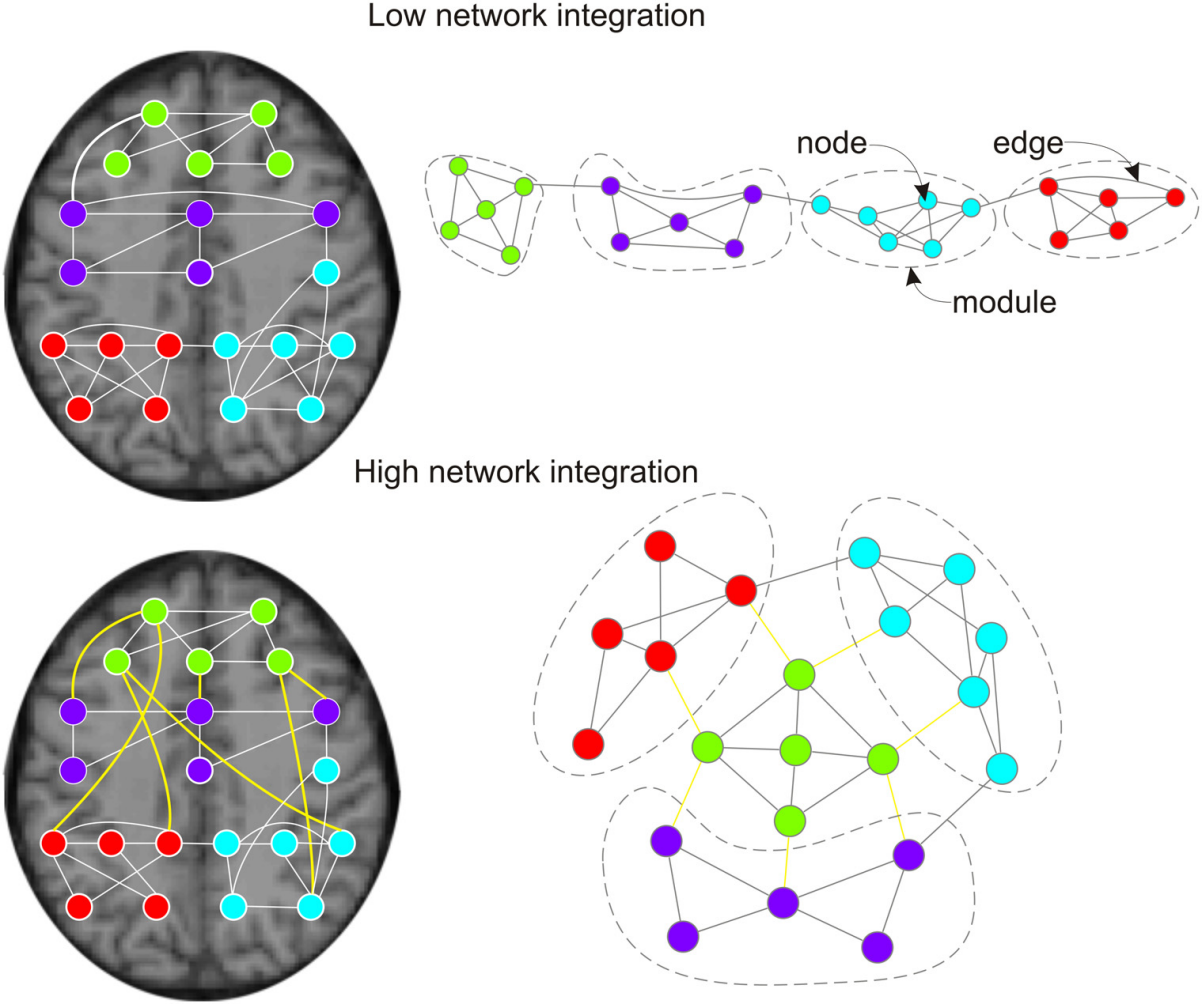
**Effects of pro-cognitive drugs on network integration in functional brain graphs.** *Left column:* functional brain graphs are constructed based on thresholded maps of functionally connected brain regions. If plotted within the physical space of the brain, functional connections between brain regions span different Euclidean distances. *Right column:* functional brain graphs describe the functional architecture of brain topology, and edges and distances between nodes reflect their functional dependencies. Thereby, functional brain graphs consist of different functional modules in which nodes show many connections (edges) to other nodes of the module, but only few edges to nodes of different functional modules. *Lower row vs. upper row:* two networks with different network integration. Previous empirical work suggest that pro-cognitive drug effects are related to increased integration of functional brain graphs, less serial processing and higher capacity for parallel information transfer. During pro-cognitive states nodes of different modules should be connected by shorter path lengths/fewer edges and brain modules are less clustered. Within the anatomical space brain modules are connected by an ensemble of functional connections with long physical distances (Alexander-Bloch et al., 2012; Vertes et al., 2012; lower left side: yellow edges). The global workspace theory of Dehaene and others (Dehaene et al., 1998; Dehaene and Naccache, 2001; Dehaene and Changeux, 2005, 2011) suggests that the pyramidal cells of the prefrontal cortex and their long cortico-cortical axons may be regarded as “workspace neurons” and play an important part in binding of different brain modules (Dehaene et al., 1998).

*Global workspace theory*: A theory first proposed by Baars (Baars, 1988, 2002, 2005) and further developed by Dehaene and others (Dehaene et al., 1998; Dehaene and Naccache, 2001) to explain the relationship between consciousness and effortful processing and brain network integration (see Figure 1 for further information).

*Graph theory*: A branch of math that analyses graphs, an abstract representation of objects where some of the objects share a relationship or interact with each other. These interactions are represented by edges and the objects are called nodes or vertices.

*Long-distance* and *short-distance connections*: In neuroscience, nodes often represent brain regions which have two different kinds of distances. Within a graph the node-to-node distance is defined by the path length or number of edges that are needed to connect two nodes. However, we use this term to describe the physical distance of brain nodes within the three-dimensional space of the scull.

*Modularity*: A system is described as modular if it can be decomposed into different subsystems or modules. These modules can have different functionality and can be recombined to match the external demands.

*Network efficiency*: In an efficient network most pairs of node are connected by a short path way. Network efficiency is also an estimator for the network's capacity for parallel information transfer.

*Network integration*: A state of a network that has low modularity and in which the mean of the shortest path ways between all pairs of nodes is small.

*Network topology*: A schematic description of the structure or layout how the objects in a network are connected or interact with each other.

*Small world networks*: A network that combines two characteristics: (1) a modular or cliquish structure that consists of highly connected sub-networks and (2) small shortest-path-lengths with few edges between nodes (Watts and Strogatz, 1998).

## Quantifying drug induced improvements of functional brain organization: network integration as performance correlate

### Empirical evidence: network integration and performance

There is strong empirical evidence that variation in network integration and network efficiency correlates with cognitive performance. This correlation has been shown, for example, for higher IQ (Hampson et al., 2006; Li et al., 2009; van den Heuvel et al., 2009) and working memory performance (Bassett et al., 2009) and shorter reaction times in a Go/NoGo task (Zhou et al., 2012a). Further support for a relationship between brain network topology and behavioral performance derives from studies which investigated the network topology in elderly volunteers. These studies found that brain networks of elderly participants have less efficient and more modular brain topology with fewer long-distance and more short-distance connections which might explain age-related performance declines in multiple cognitive functions (Achard and Bullmore, 2007; Meunier et al., 2009; Wang et al., 2010). Recently, we could also show that experimentally induced changes in task performance correlates with the efficiency of brain networks. While the cholinergic agonist nicotine improved behavioral performance in a sustained attention task and network efficiency in the resting state, time-on-task and cognitive fatigue increased reaction times and induced higher modularity within the network (Giessing et al., unpublished data; Breckel et al., unpublished data). Most of the studies reported above investigated endogenous resting state data supporting the assumption that endogenous functional brain network topology interacts with task performance and it can be assumed that endogenous functional brain network topology also interacts with task-related neural processing.

### Theoretical perspective: network integration and performance

The reported empirical correlations between functional brain network topology and behavioral performance fit with contemporary network theories of information processing in the brain, like the “brainweb” (Varela et al., 2001) or “workspace” theories (Dehaene et al., 1998; Baars, 2002). Baars et al. proposed the workspace theory of conscious perception of cognition (Baars, 1988, 2002, 2005; Shanahan and Baars, 2005) which has since been developed as global neuronal workspace theory by Dehaene and others (Dehaene and Naccache, 2001; Bartolomei and Naccache, 2011; Dehaene and Changeux, 2011). This theory predicts that conscious performance of demanding tasks requires the integration of otherwise segregated brain modules. The integration of segregated brain modules results from an ensemble of “workspace neurons” which is anatomically distributed throughout the brain (Dehaene and Naccache, 2001; Baars, 2005). It has been suggested that synchrony in the gamma band is important for the global neuronal workspace and that this gamma synchrony is regulated by GABAergic interneurons which are modulated by acetylcholine and dopamine (Changeux and Lou, 2011). Other theories like the “brainweb theory” also emphasize that behavioral performance is largely dependent on the integration of functionally specialized brain regions and that the synchronization or correlation of brain regions is a key mechanism for large-scale integration of brain networks (Varela et al., 2001). Recent evidence in humans by Bauer et al. (2012) indicated that the cholinesterase inhibitor physostigmine is able to modulate synchronization in the alpha and beta band but no evidence was found for a modulation in the gamma band. In their study, Bauer et al. (2012) have shown that the drug speeded performance and enhanced spatial attention effects on synchronization in visual cortex. In summary, the reported theories predict that higher cognitive performance goes along with higher functional integration of brain networks and at least for the cholinergic system there is first evidence that cholinergic drugs may promote the functional integration of brain networks by modulating synchronous brain activity.

### Drug effects on brain network topology

Only a few studies have used complex network analyses to investigate drug effects. We will thus mention also studies outside the cholinergic and noradrenergic system to underline the feasibility of graph analytical approaches in understanding drug effects. Complex network analyses have been previously used to partition functional brain networks into communities of densely interconnected nodes after drug challenge (Schwarz et al., 2008, 2009; Bifone et al., 2010). The authors compared dopaminergic, serotonergic and cholinergic drugs. After nicotine challenge they found two networks, one including sensorimotor cortex, thalamus, hypothalamus, hippocampus, and inferior colliculus, the other including cingulate, pre- and orbitofrontal cortex, striatum, amygdale, piriform, and entorhinal cortex as well as visual and parietal regions. For the human brain, previous results revealed that the efficiency and capacity for parallel information transfer of brain networks are affected by normal aging and by pharmacological blockade with the dopamine receptor antagonist sulpiride (Achard and Bullmore, 2007). These results suggest that the dopaminergic antagonist declines the efficiency of small world networks similar to the effects of aging. For the cholinergic system we could recently show that the cholinergic agonist nicotine increased the efficiency, reduced the modularity and clustering of brain network and drove brain functional networks to a more integrated workspace configuration (Giessing et al., unpublished data). We would assume that improvements in attention and/or cognitive control with noradrenergic drugs should similarly increase network efficiency.

Even though different drugs or other experimental conditions might have similar effects on network topology on the global, whole brain level, the underlying mechanisms might be quite different. For example, Achard and Bullmore (2007) showed that ageing and the dopamine antagonist sulpiride impaired network efficiency, but both effects were differentially localized within the brain. While the effects of ageing were found in several brain regions including the frontal and temporal neocortex, the dopamine blockade reduced efficiency mainly in the dorsal cingulate and lateral temporal cortical areas. Thus it may be reasonable to assume that even though cholinergic and noradrenergic drugs will both increase global network integration since both have pro-cognitive effects, the localization of effects may be different. In Box 2 we suggest an experimental approach to study the interaction between task-related and resting state functional connectivity in pharmacological fMRI studies.

**Measuring intermixed periods of rest and task processing—a study design for pharmacological fMRI network analyses.**

Long-lasting endogenous brain states interact with task-related processing (Li et al., 2011) and it is reasonable to assume that noradrenergic and cholinergic drugs which improve cognitive processing influence both. Unfortunately, in most paradigms the frequency range of task-induced BOLD signal changes overlaps with the frequency range (signal components below 0.1 Hz) that is analyzed in studies of endogenous processing (Achard and Bullmore, 2007). The reason for this overlap is that higher frequency inputs are more severely attenuated than low-frequency inputs by the hemodynamic response function (Friston et al., 1994, p. 163). Thus, in most paradigms it is not possible to separate endogenous activity from task processing.

**Figure 2:**
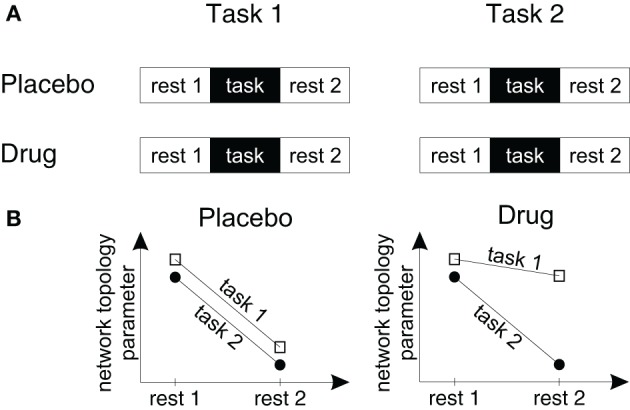
**A study design to measure drug effects on brain network topology in fMRI studies. (A)** To measure the effects of drugs, tasks and drug-by-task interactions on endogenous brain network topology we suggest a design with four different fMRI scans. Within each fMRI scan participants are measured during rest periods before and following a task block in which participants perform one of two different task conditions. Subjects are measured either following placebo or drug administration within a double blind cross-over-design. **(B)** Using this design it can be tested (1) how drugs change endogenous processing (by comparing the resting state topologies in the drug and placebo conditions), (2) whether task processing changes endogenous processing (by comparing the topology of resting state period 1 with resting state period 2), (3) whether the effects of task processing on the following resting state topology are specific for a certain task or task-independent (by pooling the drug conditions and comparing the slopes of the lines for task 1 and task 2) and (4) whether these after-effects or task-by-resting state interactions change in different drug conditions (by comparing the slopes in each drug and task condition).

To investigate drug effects on endogenous and task processing we suggest to measure both processes during different time periods (see Figure 2A). Previous results of Barnes et al. (2009) documented that task processing has long-lasting effects on resting state networks and that both interact over a time period of several minutes. This design allows to investigate the drug effects on endogenous and task-related processing. Within Figure 2B a fictional data set is illustrated with a drug-*specific* and a task-*unspecific* effect on functional resting state topology. Within the placebo condition the performance of both tasks have similar effects on the following resting state periods. However, within the drug condition only the effect of task 1 on the following resting state topology is affected.

## Future perspectives: from functional to effective connectivity graphs

Functional connectivity analyses have to deal with indirect or mediated correlations: a correlation between the time series of brain regions X1 and X2 can be mediated by a second common source X3 without any direct correlation between X1 and X2 (Erhardt et al., 2011). This problem becomes even more evident when connectivity and graph analyses are performed during task performance (Buckner et al., 2009; Wang et al., 2010; Park et al., 2012) where several brain regions react with large signal changes to external stimuli. Even though in bioinformatics sophisticated measures have been developed to distinguish direct connections from mediated connections, only few fMRI studies have used these techniques (for example Rissman et al., 2004; Marrelec et al., 2007, 2009; Schrouff et al., 2011). Future analyses of drug effects on complex networks would profit from new approaches that use complex network analysis on graphs that only represent the direct influences between neural elements.

## Clinical applications—network pharmacology

In healthy individuals as well as in many patient groups the effects of pro-cognitive drugs have been described to be modest (Husain and Mehta, 2011). However, two approaches that use functional brain network integration as a biomarker might contribute to improve pro-cognitive drug effects. First, without the need to measure behavioral performance different pharmacological substances can be screened for possible pro-cognitive drug effects in humans and animals using brain network integration during resting state periods as a biomarker. Second, effects of pro-cognitive drugs seem to be larger for individuals with lower performance levels and less optimal brain states before drug administration (Perkins, 1999; Newhouse et al., 2004; Giessing et al., 2007). Analyses of brain network topology might identify latent subgroups with less optimal brain network architecture and higher responsiveness to the cognitive enhancing drugs. Moreover, drug responsiveness can also change over the time course of a disease. Recently, it has been documented for Alzheimer's disease that network topology analyses can be used to identify the starting point, the evolution of the disease and the paths in the brain on which neurodegenerative disease spreads over the entire brain (Raj et al., 2012; Zhou et al., 2012b). This approach is an important step forward to understand the dynamics of neurodegenerative diseases. On the other side, there are new approaches to drug development, known as network pharmacology, that try to influence the entire disease-causing brain network instead of targeting specific parts of the system only (Elgoyhen et al., 2012). It will be a challenging task in the development of drugs to combine both approaches and to take the dynamic of the changing brain system into account.

On behavioral level, there is the first evidence that long-term nicotine treatment improves cognitive performance in patients with mild cognitive impairment (Newhouse et al., 2012) which in many cases has been shown to be a prodromal stage of Alzheimer's disease (Petersen, 2000a,b; Grundman et al., 2004). This is also reflected in changes in network topology which are intermediate between healthy controls and Alzheimer's patients (Yao et al., 2010). It is an open question whether nicotine-induced changes in behavior in patients with mild cognitive impairment prevent further dysfunctional changes in network topology.

### Conflict of interest statement

The authors declare that the research was conducted in the absence of any commercial or financial relationships that could be construed as a potential conflict of interest.
